# Vitamin D: a 14-year retrospective study at a clinical laboratory in Brazil

**DOI:** 10.20945/2359-3997000000427

**Published:** 2022-01-14

**Authors:** Fernanda Vaz de Melo Bacha, Fernanda Lustosa Cabral Gomez, Ana Luiza Gonçalves Silva, Mariana Didier Reis, Eliane Dias Lustosa Cabral, Leandro Duarte de Carvalho

**Affiliations:** 1 Faculdade Ciências Médicas de Minas Gerais Belo Horizonte MG Brasil Faculdade Ciências Médicas de Minas Gerais (FCMMG), Belo Horizonte, MG, Brasil; 2 Centro de Desenvolvimento, Inovação, Ciência e Tecnologia Lagoa Santa MG Brasil Centro de Desenvolvimento, Inovação, Ciência e Tecnologia/Labtest Diagnóstica S.A., Lagoa Santa, MG, Brasil

**Keywords:** Vitamin D, vitamin D deficiency, sex, age groups, Brazil

## Abstract

**Objective:**

This study aimed to assess vitamin D (25OHD) levels in individuals who underwent an examination at a private laboratory (between latitudes 14° and 22° south) over 14 years, stratified by sex, age, and epidemiological profiles, and determine variations in the number of tests performed over the years.

**Materials and methods:**

All records of 25OHD tests performed at a private clinical laboratory in Brazil were analyzed. This retrospective cross-sectional study included patients stratified by sex (female or male), age range (0-17, 18-40, 41-59, and ≥ 60 years), and year of testing. The final sample size was 193,725 patients. Categorical variables are presented as absolute and relative frequencies and numerical variables as means ± standard deviation. Comparisons between groups were performed using the equality of proportions test.

**Results:**

The number of tests performed steeply increased since 2010. More tests were performed in female individuals (73.3%) and individuals aged 41-59 years (32.2%). Most samples (68.0%) demonstrated sufficient vitamin D status. Women had a higher incidence of vitamin D deficiency than men (33.1% and 26.6%, respectively; p < 0.001). Individuals aged ≥ 60 years had the highest incidence of vitamin D deficiency (68.4%), while individuals aged 0-17 years had the lowest (32.2%) (p < 0.001).

**Conclusion:**

Despite increased testing and attention given to vitamin D in recent years, our study demonstrates high levels of deficiency in a country with geographical conditions favorable to its production.

## INTRODUCTION

The main role of vitamin D (25OHD) is to modulate the synthesis of parathyroid hormone, optimize intestinal calcium absorption, and aid in bone and muscle homeostasis. It also has several non-classical functions – in immunological processes, growth and cell differentiation, hormone production, and blood pressure control. Considering the functional range of this pre-hormone, deviations in its serum levels are associated with clinically significant pathophysiologies (1,2).

Vitamin D deficiency is primarily related to conditions such as hyperparathyroidism and musculoskeletal diseases, including rickets, osteomalacia, and muscle weakness. It is also involved in the development of cardiovascular diseases, autoimmune diseases, and neoplasms such as colon and prostate cancers (2). With regard to the coronavirus disease pandemic, several studies are evaluating a possible association between vitamin D deficiency and the evolution of the disease. Although patients with the disease have vitamin D deficiency, there is not yet sufficient evidence to support the use of vitamin D in its treatment (3). Serum 25OHD depend on its ingestion, as well as formation from a precursor in the skin by sun exposure. Therefore, its level is influenced by factors such as diet, exposure to ultraviolet radiation, time of the year, latitude, skin pigmentation, age, and use of sunscreens (1,4).

The prevalence of vitamin D deficiency is increasing worldwide. Despite the difficulty in comparing the results of different studies due to the diversity among individuals and the different the cut-off points used to define deficiency, low levels of this hormone have been reported in the general population (1,2). A study analyzing the prevalence of vitamin D in the North American population, which included over 25,000 individuals, found a deficiency in 28.9% of patients (1). A European study of 55,000 participants reported a deficiency in 40% of patients. In Mexico, the rate was 37% in the older population (2). A Brazilian meta-analysis, comparing the results of 72 studies, found the prevalence of vitamin D deficiency to be 28.1%, contradicting the idea that countries with high sun exposure have lower rates of vitamin D deficiency (4). The reference value for deficiency was set at 50 nmol/L (20 ng/mL) in all these studies. Results of studies on the real impact of this deficiency on the population remain inconclusive.

The purpose of the present study was to analyze the vitamin D status and the epidemiological profiles of individuals who underwent testing at a private laboratory over 14 years. In addition, we aimed to stratify vitamin D status by gender and age group and establish the prevalence of its deficiency in the studied population. We also planned to enumerate the number of tests performed over the years.

## MATERIALS AND METHODS

This was a sectional study that assessed all records of 25OHD serum concentration tests performed at a private clinical laboratory in Brazil (located between 14° and 22° south latitudes). The Research Ethics Committee of *Faculdade de Ciências Médicas de Minas Gerais* approved this study (FCMMG; CAAE: 76801917.3.0000.5134). Explicit consent was waived by the ethics committee because of the retrospective nature of the study. All data were de-identified prior to analysis.

### Study population

The inclusion criterion was all patients who underwent 25OHD testing at the laboratory between January 01, 2004 and December 1, 2018. Patients without identification of sex or age, and those whose tests has undefined results, were excluded. The total number of patients was 194,010 and the total number of tests was 492,540. After excluding 285 patients and 385 results, 193,725 eligible patients and 492,155 test results remained. In patients who underwent more than one test, the first results were used to avoid the interference of treatment. In total, 193,725 tests were analyzed in this study.

### Analytical methods

The laboratory where the participants of this study were tested utilized different methods to assess vitamin D status over the years. Until 2013, tests were performed in a support laboratory; hence, it was not possible to establish the method used. From 2013 till date, chemiluminescence was used, although the reagents and equipment were from different brands: Diasorin/Liaison from 2013 to 2016, Abbott/Architect from 2016 to 2017, and Siemens/Centaur from 2017 to 2018.

In this study, the cut-off points for vitamin D status were defined according to the guidelines of the 2018 update of the Brazilian Society of Endocrinology and Metabolism (SBEM) in association with the Brazilian Society of Clinical Pathology/Laboratory Medicine (SBPC/ML). In individuals aged < 60 years, values < 20 ng/mL were considered deficient, while those of 20-60 ng/mL were considered sufficient. In individuals aged ≥ 60 years, deficiency was defined as values < 30 ng/mL and sufficiency as values between 30 and 60 ng/mL. In both groups, vitamin D status > 100 ng/mL represented a risk of hypervitaminosis or toxicity (5,6). For data analysis, all the 25OHD records were stratified by sex (female or male), age range (0-17 years, 18-40 years, 41-59 years, and ≥ 60 years), and the year in which the test was performed, for comparison of vitamin D status between the groups.

### Statistical analysis

The laboratory operating system “SHIFT” was used to stratify the data by gender, age range, and year of testing. Categorical variables were presented as absolute and relative frequencies, and numerical variables as mean ± standard deviation. The comparison between the two groups was performed using the equality of proportions test. The level of significance was set at 5%, and the data were analyzed using R version 4.0.3 software.

## RESULTS

### Testing demographics

**Figure 1:**
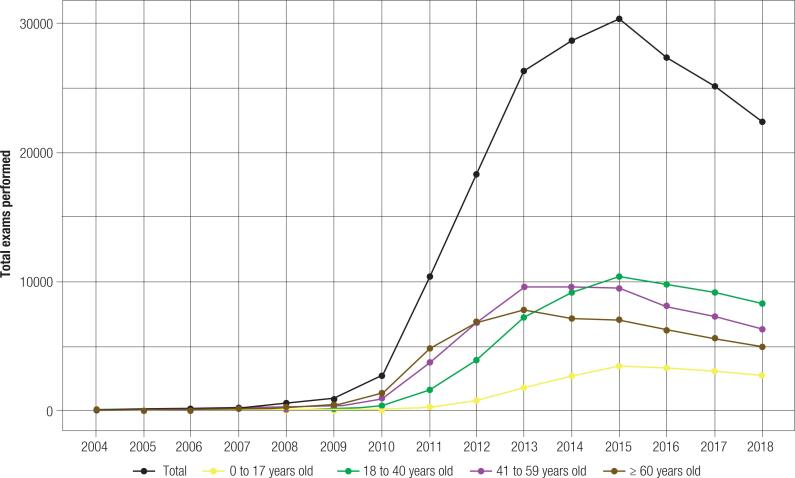
Total tests performed over time stratified by age range.

The final sample included 193,725 patients, with 142,052 (73.3%) women. The 41-59 years age group had the highest number of tests (62,412; 32.2%), followed by the 18-40 years group (60,192; 31.1%), the ≥ 60 years group (52,920; 27.3%), and the 0-17 years group (18,201; 9.4%). The mean age of the patients was 46.2 ± 20.7 years. As shown in Figure 1, the age range with most examinations performed per year changed in 2015. From 2015 to 2018, patients aged 18-40 years had the highest number of tests per year. The total number of tests performed increased over time in all age ranges, with an abrupt growth in 2010.

### Test results

The mean concentration of 25OHD was 27.4 ± 13.5 ng/mL. For females, it was 26.9 ± 14.6 ng/mL, while for males, it was 28.8 ± 9.7 ng/mL. The mean concentration of 25OHD in the age groups of 0-17 years, 18-40 years, 41-59 years, and ≥ 60 years were 29.7 ± 9.0 ng/mL, 27.9 ± 9.7 ng/mL, 27.0 ± 9.8 ng/mL, and 26.4 ± 20.4 ng/mL, respectively (as shown in Tables 1 and S1).

**Table 1:** Classification of vitamin D status stratified by age range

		Age Range			
		0-17 years	18-40 years	41-59 years	≥60 years
Total					
Mean level (ng/mL)		29.7 ± 9.0	27.9 ± 9.7	27.0 ± 9.8	26.4 ± 20.4
	Deficient (n/%)	1,910 (10.5)	10,210 (17.0)	12,363 (19.8)	36,221 (68.4)
	Sufficient (n/%)	16,177 (88.9)	49,591 (82.4)	49,707 (79.6)	16,308 (30.8)
	≥60 to 100 (n/%)	108 (0.6)	327 (0.5)	266 (0.4)	315 (0.6)
	Intoxication (n/%)	6 (0.0)	64 (0.1)	76 (0.1)	76 (0.1)

**Figure 2:**
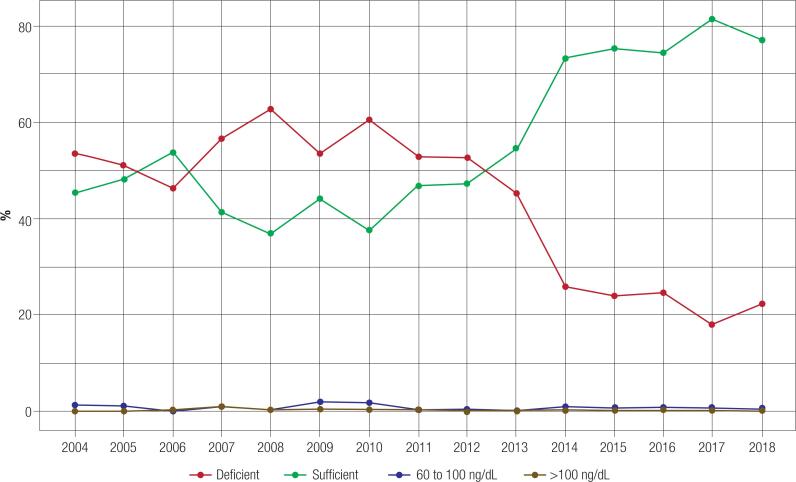
Percentage of vitamin D status for all patients over time.

The test results showed that sufficient levels of 25OHD were predominantly seen (68.0%), followed by deficient levels (31.3%), < 10 ng/mL (1,4%), ≥ 60 to 100 ng/mL (0.5%), and intoxication (0.1%). When stratified by age range, sufficient vitamin D status were seen in all age groups except the ≥ 60 years group, as shown in Tables 1 and S1. Until 2010, the elderly individuals were responsible for most of the tests performed as well as for most of the deficiencies seen in the studied population. As shown in Figure 2, the total number of patients with deficiency was less than the total number of those with sufficiency after 2013.

**Figure 3:**
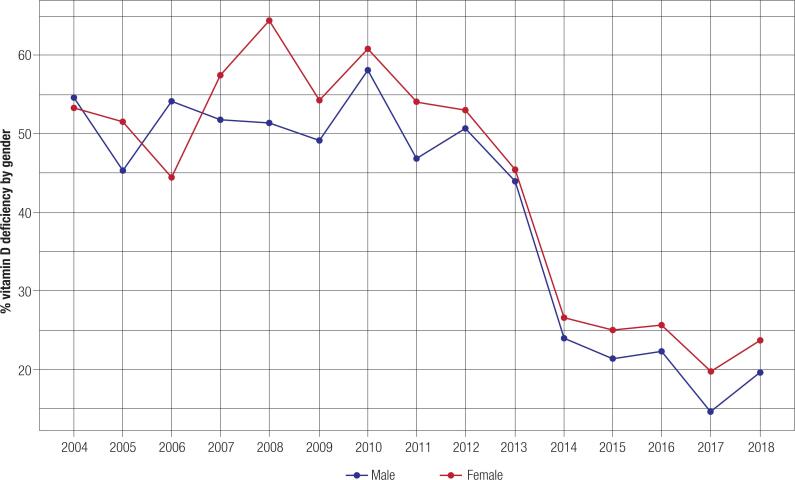
Proportion of patients with vitamin D deficiency stratified by gender.

The percentage of females and males with vitamin D deficiency were 33.1% and 26.6%, respectively. Overall, women had significantly lower levels than men (p < 0.001). Tables 2 and S2 shows the concentration of 25OHD in different age groups in both sexes. A predominance of sufficiency in those aged < 60 years and deficiency in those aged ≥ 60 years can be noted. Until 2012, the high number of individuals aged ≥ 60 years influenced the trend of deficiencies. As shown in Figure 3, from 2013, the influence of the female to male proportion among elderly individuals was corrected. From 2014 to 2018, women had significantly lower levels of vitamin D than men (p < 0.001). In 2008 and 2012, there was a significant difference in the levels between men and women (p = 0.038 and p = 0.014, respectively). In addition, women had significantly more prevalence of deficiency in all age groups (p < 0.001).

**Table 2:** Classification of vitamin D status stratified by age range and gender

		<60 years		≥60 years	
		Female	Male	Female	Male
Total					
Mean level (ng/mL)		27.2 ± 9.7	29.2 ± 9.6	25.9 ± 23.2	28.0 ± 9.9
	Deficient (n/%)	19,851 (19.1)	4,632 (12.5)	27,112 (70.9)	9,109 (62.0)
	Sufficient (n/%)	83,401 (80.3)	32,074 (86.7)	10,837 (28.4)	5,471 (37.2)
	≥60 to 100 (n/%)	472 (0.5)	229 (0.6)	214 (0.6)	101 (0.7)
	Intoxication (n/%)	103 (0.1)	43 (0.1)	62 (0.2)	14 (0.1)

**Figure 4:**
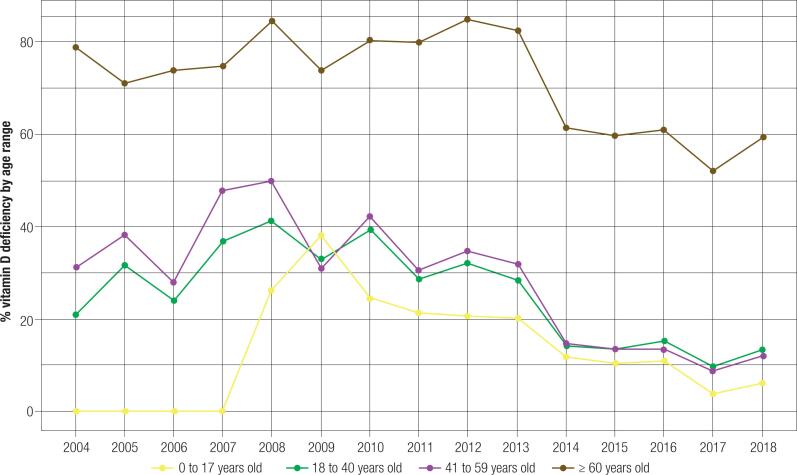
Proportion of patients with vitamin D deficiency stratified by age range.

When stratified by age ranges, the groups with the lowest vitamin D status were ≥ 60 years old (68.4%), followed by 41-59 years (19.8%), 18-40 years (17%), and 0-17 years (10.5%) (p < 0.001) (Tables 1 and S1). The highest number of patients with vitamin D deficiency among all age groups was seen in the ≥ 60 years group (p < 0.05). This became more significant in 2010 and all subsequent years (p < 0.001). Throughout the study period, the group aged 0-17 years was the least deficient (p < 0.05 until 2012 and p < 0.001 from 2012 to 2018). In all years, the elderly patients had mostly deficient vitamin D status, whereas the other age ranges had predominantly sufficient levels (Figure 4).

Given that the high proportion of individuals aged ≥ 60 years with vitamin D deficiency was interfering with the overall result, we used a cut-off value of 20 ng/mL to assess whether patients aged ≥ 60 years really had such a high degree of deficiency or if the higher cut-off was leading to overestimation of the deficiency. The results indicated that individuals aged ≥ 60 years had more deficient levels than those aged < 60 years (25.2% and 17.4%, respectively; p < 0.001). With this change, vitamin D deficiency was present in 19.5% of the final sample.

Our study also indicated that there are variations in serum 25OHD according to seasonality. Mean serum 25OHD in the Summer, Autumn, Winter and Spring were 29.4 ± 21.4, 28.5 ± 10.4, 25.3 ± 9.2 and 26.5 ± 9.7, respectively (p < 0,001). Meanwhile, deficient serum 25OHD was found in 25.3%, 27.2%, 37.9% and 34.2%, respectively (p < 0,001).

## DISCUSSION

### Testing demographics

In this retrospective study conducted at a private laboratory in Brazil, we were able to identify an increase in laboratory test requests over time, with an abrupt growth since 2010. This result is similar to other studies conducted in countries such as Australia, France, Saudi Arabia, the United States of America, and the United Kingdom (7-12). An increasing amount of data suggesting an association between vitamin D and the prevention and treatment of skeletal muscle problems, cardiovascular system diseases, and autoimmune diseases can partially explain this phenomenon (13). Reinforcing this trend, the PubMed search platform showed 11,415 new articles related to vitamin D that were published between 2004 and 2009, whereas from 2010 to 2015, 22,384 new publications were released. In addition, bigger announcement by the media at this time made the information more accessible, even to non-specialists (14).

With the increasing demand for 25OHD testing, in 2017 the SBEM in association with SBPC/ML suggested that there should be formal indications for its solicitation. According to these societies, this test is indicated in the elderly (above 60 years); individuals with low sun exposure; individuals with recurrent fractures or falls; pregnant or nursing women; individuals with primary or secondary osteoporosis; individuals with osteometabolic diseases such as osteomalacia, hyperparathyroidism, and rickets; individuals with chronic renal disease; individuals with malabsorption syndromes such as post-bariatric surgery syndrome and inflammatory bowel disease; and individuals who take medications that interfere with the formation or degradation of vitamin D (such as antiretroviral therapy, glucocorticoids, and anticonvulsant drugs). Thus, indiscriminate screening of the population is not recommended (6).

The higher prevalence of testing among females (73%) is similar to the results found in studies by Zhao and cols. (12) and Woodford and cols. (7), which demonstrated that the prevalence of testing in males was 25% and 30%, respectively. These findings are consistent with the fact that women are usually more careful about their health and body (15).

Most examinations were performed in the 41-59 years group (32.2%), followed by the 18-40 years group (31.1%). These numbers are somewhat similar to those reported by Woodford and cols. (7) who revealed that 40% of tests were performed in individuals aged 30-60 years and by Zhao and cols. (12) who revealed that the mean age of patients was 50 years. The age groups with the highest prevalence had increased requests for testing during the ascension period in 2010.

### Test results

This study shows that in the analyzed period, 68% of the participants had sufficient vitamin D status while 31.3% had deficient levels. The mean vitamin D status in the study population was 27.4 ± 13.5 ng/mL. These results correspond to the values from the national meta-analysis; Pereira-Santos and cols. (4) which described a 28.16% prevalence of vitamin D deficiency in the Brazilian population, with an even higher percentage in the southeast region of the country (31.11%). Furthermore, the mean vitamin D status in the same meta-analyses was 27.06 ng/mL in the whole population, and 27.09 ng/dL in the southeast region. It is worth noting that the present study was conducted in the southeastern region of Brazil.

Studies conducted in places at higher latitudes, such as Canada and Europe, demonstrate an even greater prevalence of vitamin D deficiency (37% and 40%, respectively) (16,17). However, a study conducted in the USA (a place at higher latitude compared to Brazil) reported a 24% prevalence of vitamin D deficiency, which is lower than that in Brazil (18). Hilger and cols. (19) claimed that the deficiency levels previously described in Brazilian studies are similar to those found in countries with high latitudes that have less sun exposure, and as a result, less vitamin D formation in the skin (20,21). However, it is possible that the use of sunscreen, pollution, and low intake of vitamin D contribute to similar levels of this nutrient in countries with less UV incidence (20,22). Another aspect is the lack of a standard cut-off between countries (2). In line with our findings, several studies also indicate that there are variations in serum 25OHD according to seasonality; this is due to the greater importance of skin production through UV exposure (1,4,23).

In this study, prior to 2013, the population analyzed was mostly deficient. From 2013, the examination results were found to be predominantly sufficient. As previously mentioned, the proportion of deficiency before 2013 was strongly influenced by the fact that the majority of the population was elderly. In 2014, the percentage of deficiency in all groups was reduced by approximately 25%. Between 2013 and 2014, there was a significant difference in the proportion of vitamin D deficiency for women and men, both aged under 60 years and over 60 years (all p-values were < 0.001).

The publication of the article “Recommendations of the Brazilian Society of Endocrinology and Metabolism for the diagnosis and treatment of hypovitaminosis D” in 2014, which encouraged vitamin D supplementation, is a potential explanation for the growth in the number of patients with sufficient vitamin D status since that year. Another possible reason is the change in the method and reagents used to process the test. Prior to 2013, the analysis was outsourced by the laboratory in question due to relatively low demand. Thus, it was not possible to determine the methodology employed during that period. In 2013, the laboratory implemented high sensibility parameters, as they used the chemiluminescence method from Diasorin, Abbot, and Siemens (24). It is possible that the methods used before 2013 underestimated the results, as they had lower sensitivity (25-27).

The prevalence of vitamin D deficiency was higher in women than in men (p < 0.001). This result is in agreement with the direct association between endogenous testosterone secretion and vitamin D status (28,29). In contrast, Eloi and cols. (30) in a study conducted in São Paulo with 39,004 individuals aged 2-92 years, identified that there was no significant difference in vitamin D status between sexes. In the Brazilian literature, there are not many studies comparing the genders, while in international studies, gender predilection changes with the location and its environmental, social, and cultural aspects (31,32). In all four age groups, vitamin D deficiency was significantly higher in the female population (p < 0.001). This outcome was also described by Lima-Costa and cols. (33) and de Oliveira and cols. (34) in their studies on the elderly and adolescents aged 12-17 years, respectively.

Individuals aged ≥ 60 years were the most deficient in vitamin D status (68.4%), while those aged 0-17 years were the least deficient (10.5%) (p < 0.001). These data agree with the national meta-analysis developed by Pereira-Santos and cols. (4), which showed a deficiency in 41.53% of the elderly, 35.73% of adults, and 14.50% and 22.95% of adolescents and children, respectively. The vitamin D status considered deficient in the meta-analyses was different from that recommended by the SBEM in 2018; hence, it differs from the cut-off point used in this study. Greater deficiency in the elderly is related to less sun exposure and lower capacity of dermal vitamin D production (35).

Finally, even after reducing the cut-off used for those aged ≥ 60 years to 20 ng/mL, vitamin D deficiency was still more prevalent in the older age group. A threshold of 30 ng/mL has been shown to be more adequate for individuals in the high-risk group, since it is known that vitamin D status of 10-20 ng/mL are related to increased bone remodeling, loss of bone mass, risk of fracture, and osteoporosis; this risk is already higher in the elderly (5).

This study has potential limitations. As a laboratory-based retrospective study we could be facing selection bias. Also, there is no information regarding the indication for the exam, as well as the use of supplements.

In conclusion, the number of 25OHD tests requested over the years has grown abruptly in all genders and age groups. Females were tested the most and had a higher proportion of deficiency between genders in all age groups. The elderly (≥60 years) had a higher prevalence of deficiency, while most individuals aged 0-17 years had sufficient levels. Despite the increase in tests performed and the attention given to vitamin D in recent years, data still show an elevated rate of deficiency in a country with propitious geographic conditions for its production.

## SUPPLEMENTARY TABLES

### Table S1 Classification of vitamin D status stratified by age range

**Table:**

Years		Age Range				
		0-17 years	18-40 years	41-59 years	≥60 years	P^K^
**2004**						
Mean level (ng/mL)		33.0	28.5 ± 12.1	25.7 ± 10.4	22.9 ± 9.9	0.197
	Deficient (n/%)	0 (0.0)	5 (20.8)	5 (31.2)	37 (78.7)	
	Sufficient (n/%)	1 (100.0)	18 (75.0)	11 (68.8)	10 (21.3)	
	≥60 to 100 (n/%)	0 (0.0)	1 (4.2)	0 (0.0)	0 (0.0)	
	Intoxication (n/%)	0 (0.0)	0 (0.0)	0 (0.0)	0 (0.0)	
**2005**						
Mean level (ng/mL)		25.8	26.5 ± 10.7	24.6 ± 10.5	25.3 ± 10.1	0.863
	Deficient (n/%)	0 (0.0)	6 (31.6)	16 (38.1)	34 (70.8)	
	Sufficient (n/%)	1 (100.0)	13 (68.4)	26 (61.9)	13 (27.1)	
	≥60 to 100 (n/%)	0 (0.0)	0 (0.0)	0 (0.0)	1 (2.1)	
	Intoxication (n/%)	0 (0.0)	0 (0.0)	0 (0.0)	0 (0.0)	
**2006**						
Mean level (ng/mL)		28.0 ± 0.0	27.4 ± 8.7	26.6 ± 10.5	23.8 ± 9.7	0.177
	Deficient (n/%)	0 (0.0)	5 (23.8)	15 (27.8)	42 (73.7)	
	Sufficient (n/%)	2 (100.0)	16 (76.2)	39 (72.2)	15 (26.3)	
	≥60 to 100 (n/%)	0 (0.0)	0 (0.0)	0 (0.0)	0 (0.0)	
	Intoxication (n/%)	0 (0.0)	0 (0.0)	0 (0.0)	0 (0.0)	
**2007**						
Mean level (ng/mL)		32.0 ± 8.4	24.4 ± 9.5	23.4 ± 11.0	24.0 ± 16.9	0.053
	Deficient (n/%)	0 (0.0)	11 (36.7)	32 (47.8)	68 (74.7)	
	Sufficient (n/%)	8 (100.0)	19 (63.3)	34 (50.7)	20 (22.0)	
	≥60 to 100 (n/%)	0 (0.0)	0 (0.0)	1 (1.5)	1 (1.1)	
	Intoxication (n/%)	0 (0.0)	0 (0.0)	0 (0.0)	2 (2.2)	
**2008**						
Mean level (ng/mL)		28.7 ± 11.0	24.7 ± 12.0	21.0 ± 9.2	20.6 ± 9.1	<0.001
	Deficient (n/%)	5 (26.3)	40 (41.2)	103 (49.8)	212 (84.5)	
	Sufficient (n/%)	14 (73.7)	56 (57.7)	104 (50.2)	37 (14.7)	
	≥60 to 100 (n/%)	0 (0.0)	1 (1.0)	0 (0.0)	1 (0.4)	
	Intoxication (n/%)	0 (0.0)	0 (0.0)	0 (0.0)	1 (0.4)	
**2009**						
Mean level (ng/mL)		27.0 ± 11.5	27.7 ± 15.2	27.0 ± 13.3	24.4 ± 14.2	0.002
	Deficient (n/%)	8 (38.1)	35 (32.7)	95 (31.0)	345 (73.7)	
	Sufficient (n/%)	13 (61.9)	67 (62.6)	207 (67.6)	110 (23.5)	
	≥60 to 100 (n/%)	0 (0.0)	5 (4.7)	3 (1.0)	11 (2.4)	
	Intoxication (n/%)	0 (0.0)	0 (0.0)	1 (0.3)	2 (0.4)	
**2010**						
Mean level (ng/mL)		26.0 ± 10.3	26.5 ± 14.4	24.0 ± 11.6	23.1 ± 14.1	<0.001
	Deficient (n/%)	12 (24.5)	135 (39.5)	402 (42.1)	1,074 (80.2)	
	Sufficient (n/%)	36 (73.5)	197 (57.6)	541 (56.6)	237 (17.7)	
	≥60 to 100 (n/%)	1 (2.0)	9 (2.6)	11 (1.2)	24 (1.8)	
	Intoxication (n/%)	0 (0.0)	1 (0.3)	2 (0,2)	4 (0.3)	
**2011**						
Mean level (ng/mL)		26.4 ± 7.8	25.4 ± 9.9	24.9 ± 9.5	23.4 ± 10.2	<0.001
	Deficient (n/%)	56 (21.3)	461 (28.7)	1,132 (30.6)	3,827 (79.8)	
	Sufficient (n/%)	207 (78.7)	1,138 (70.9)	2,555 (69.0)	941 (19.6)	
	≥60 to 100 (n/%)	0 (0.0)	5 (0.3)	12 (0.3)	14 (0.3)	
	Intoxication (n/%)	0 (0.0)	2 (0.1)	5 (0.1)	13 (0.3)	
**2012**						
Mean level (ng/mL)		26.1 ± 8.0	24.0 ± 8.0	23.2 ± 7.8	22.2 ± 8.9	<0.001
	Deficient (n/%)	151 (20.5)	1,244 (32.2)	2,354 (34.7)	5,878 (84.8)	
	Sufficient (n/%)	582 (79.2)	2,613 (67.6)	4,421 (65.2)	1,034 (14.9)	
	≥60 to 100 (n/%)	2 (0.3)	5 (0.1)	7 (0.1)	17 (0.2)	
	Intoxication (n/%)	0 (0.0)	2 (0.1)	2 (0.0)	6 (0.1)	
**2013**						
Mean level (ng/mL)		25.9 ± 7.4	24.7 ± 8.3	23.6 ± 7.6	23.0 ± 8.6	<0.001
	Deficient (n/%)	359 (20.1)	2,058 (28.4)	3,043 (31.9)	6,426 (82.3)	
	Sufficient (n/%)	1,421 (79.7)	5,173 (71.4)	6,474 (67.9)	1,352 (17.3)	
	≥60 to 100 (n/%)	2 (0.1)	16 (0.2)	14 (0.1)	19 (0.2)	
	Intoxication (n/%)	0 (0.0)	2 (0.0)	6 (0.1)	8 (0.1)	
**2014**						
Mean level (ng/mL)		29.0 ± 8.6	28.9 ± 9.3	28.3 ± 9.0	28.3 ± 9.7	<0.001
	Deficient (n/%)	320 (11.7)	1,287 (14.0)	1,417 (14.8)	4,408 (61.4)	
	Sufficient (n/%)	2,411 (87.8)	7,816 (85.2)	8,082 (84.5)	2,712 (37.8)	
	≥60 to 100 (n/%)	14 (0.5)	68 (0.7)	57 (0.6)	52 (0.7)	
	Intoxication (n/%)	0 (0.0)	7 (0.1)	10 (0.1)	9 (0.1)	
**2015**						
Mean level (ng/mL)		29.5 ± 8.7	29.1 ± 9.5	28.7 ± 9.1	28.7 ± 10.0	<0.001
	Deficient (n/%)	355 (10.3)	1,427 (13.7)	1,277 (13.5)	4,189 (59.4)	
	Sufficient (n/%)	3,066 (89.1)	8,877 (85.4)	8,143 (85.9)	2,800 (39.7)	
	≥60 to 100 (n/%)	19 (0.6)	82 (0.8)	44 (0.5)	53 (0.8)	
	Intoxication (n/%)	0 (0.0)	12 (0.1)	17 (0.2)	11 (0.2)	
**2016**						
Mean level (ng/mL)		30.1 ± 10.0	28.9 ± 9.9	28.8 ± 9.6	28.8 ± 10.1	<0.001
	Deficient (n/%)	350 (10.7)	1,497 (15.3)	1,080 (13.4)	3,807 (60.9)	
	Sufficient (n/%)	2,876 (88.3)	8,196 (83.8)	6,915 (85.7)	2,381 (38.1)	
	≥60 to 100 (n/%)	28 (0.9)	80 (0.8)	55 (0.7)	59 (0.9)	
	Intoxication (n/%)	4 (0.1)	13 (0.1)	16 (0.2)	4 (0.1)	
**2017**						
Mean level (ng/mL)		32.2 ± 8.2	29.0 ± 10.4	29.3 ± 9.7	29.5 ± 9.5	<0.001
	Deficient (n/%)	118 (3.9)	882 (9.6)	640 (8.8)	2,921 (51.9)	
	Sufficient (n/%)	2,910 (95.3)	8,272 (90.0)	6,615 (90.7)	2,671 (47.5)	
	≥60 to 100 (n/%)	23 (0.8)	23 (0.3)	33 (0.5)	25 (0.4)	
	Intoxication (n/%)	2 (0.1)	14 (0.2)	9 (0.1)	7 (0.1)	
**2018**						
Mean level (ng/mL)		31.4 ± 8.9	27.9 ± 9.9	28.5 ± 13.1	29.9 ± 58.3	<0.001
	Deficient (n/%)	176 (6.2)	1,117 (13.5)	752 (11.9)	2,953 (59.4)	
	Sufficient (n/%)	2,629 (93.1)	7,120 (86.0)	5,540 (87.5)	1,975 (39.7)	
	≥60 to 100 (n/%)	19 (0.7)	32 (0.4)	29 (0.5)	38 (0.8)	
	Intoxication (n/%)	0 (0.0)	11 (0.1)	8 (0.1)	9 (0.2)	
**Total**						
Mean level (ng/mL)		29.7 ± 9.0	27.9 ± 9.7	27.0 ± 9.8	26.4 ± 20.4	<0.001
	Deficient (n/%)	1,910 (10.5)	10,210 (17.0)	12,363 (19.8)	36,221 (68.4)	
	Sufficient (n/%)	16,177 (88.9)	49,591 (82.4)	49,707 (79.6)	16,308 (30.8)	
	≥60 to 100 (n/%)	108 (0.6)	327 (0.5)	266 (0.4)	315 (0.6)	
	Intoxication (n/%)	6 (0.0)	64 (0.1)	76 (0.1)	76 (0.1)	

k Kruskal-Wallis test

### Table S2 Classification of vitamin D status stratified by age range and gender

**Table:**

Years		<60 years			≥60 years		
		Female	Male	P^M^	Female	Male	P^M^
**2004**							
	Mean level (ng/mL)	28.4 ± 11.4	20.7 ± 8.8	0.152	22.9 ± 10.1	23.0 ± 9.1	0.799
	Deficient (n/%)	8 (22.2)	2 (40.0)		33 (80.5)	4 (66.7)	
	Sufficient (n/%)	27 (75.0)	3 (60.0)		8 (19.5)	2 (33.3)	
	≥60 to 100 (n/%)	1 (2.8)	0 (0.0)		0 (0.0)	0 (0.0)	
	Intoxication (n/%)	0 (0.0)	0 (0.0)		0 (0.0)	0 (0.0)	
**2005**							
	Mean level (ng/mL)	25.1 ± 10.6	25.9 ± 10.1	0.941	25.0 ± 10.4	29.5 ± 4.4	0.201
	Deficient (n/%)	19 (35.2)	3 (37.5)		32 (71.1)	2 (66.7)	
	Sufficient (n/%)	35 (64.8)	5 (62.5)		12 (26.7)	1 (33.3)	
	≥60 to 100 (n/%)	0 (0.0)	0 (0.0)		1 (2.2)	0 (0.0)	
	Intoxication (n/%)	0 (0.0)	0 (0.0)		0 (0.0)	0 (0.0)	
**2006**							
	Mean level (ng/mL)	26.8 ± 8.7	27.1 ± 13.5	0.674	24.3 ± 10.2	20.8 ± 5.5	0.490
	Deficient (n/%)	14 (23.0)	6 (37.5)		35 (71.4)	7 (87.5)	
	Sufficient (n/%)	47 (77.0)	10 (62.5)		14 (28.6)	1 (12.5)	
	≥60 to 100 (n/%)	0 (0.0)	0 (0.0)		0 (0.0)	0 (0.0)	
	Intoxication (n/%)	0 (0.0)	0 (0.0)		0 (0.0)	0 (0.0)	
**2007**							
Mean level (ng/mL)		24.0 ± 10.4	25.6 ± 11.2	0.582	24.2 ± 17.2	21.7 ± 14.0	0.613
	Deficient (n/%)	34 (40.0)	9 (45.0)		63 (75.0)	5 (71.4)	
	Sufficient (n/%)	50 (58.8)	11 (55.0)		18 (21.4)	2 (28.6)	
	≥60 to 100 (n/%)	1 (1.2)	0 (0.0)		1 (1.2)	0 (0.0)	
	Intoxication (n/%)	0 (0.0)	0 (0.0)		2 (2.4)	0 (0.0)	
**2008**							
Mean level (ng/mL)		22.1 ± 10.2	25.7 ± 11.7	0.041	20.3 ± 8.8	22.7 ± 11.1	0.356
	Deficient (n/%)	130 (46.9)	18 (39.1)		191 (86.4)	21 (70.0)	
	Sufficient (n/%)	147 (53.1)	27 (58.7)		28 (12.7)	9 (30.0)	
	≥60 to 100 (n/%)	0 (0.0)	1 (2.2)		1 (0.5)	0 (0.0)	
	Intoxication (n/%)	0 (0.0)	0 (0.0)		1 (0.5)	0 (0.0)	
**2009**							
Mean level (ng/mL)		26.9 ± 13.7	29.1 ± 13.8	0.205	24.0 ± 14.2	27.2 ± 14.1	0.047
	Deficient (n/%)	122 (32.4)	16 (28.1)		302 (74.6)	43 (68.3)	
	Sufficient (n/%)	248 (65.8)	39 (68.4)		92 (22.7)	18 (28.6)	
	≥60 to 100 (n/%)	6 (1.6)	2 (3.5)		9 (2.2)	2 (3.2)	
	Intoxication (n/%)	1 (0.3)	0 (0.0)		2 (0.5)	0 (0.0)	
**2010**							
Mean level (ng/mL)		24.4 ± 12.0	26.5 ± 14.9	0.051	22.9 ± 13.9	24.5 ± 14.8	0.054
	Deficient (n/%)	491 (41.5)	58 (35.2)		913 (81.0)	161 (75.9)	
	Sufficient (n/%)	669 (56.6)	105 (63.6)		190 (16.9)	47 (22.2)	
	≥60 to 100 (n/%)	20 (1.7)	1 (0.6)		21 (1.9)	3 (1.4)	
	Intoxication (n/%)	2 (0.2)	1 (0.6)		3 (0.3)	1 (0.5)	
**2011**							
Mean level (ng/mL)		24.8 ± 9.3	26.7 ± 10.5	<0.001	23.1 ± 10.3	24.9 ± 9.4	<0.001
	Deficient (n/%)	1,445 (30.9)	204 (22.6)		3,232 (81.0)	595 (74.1)	
	Sufficient (n/%)	3,206 (68.6)	694 (77.0)		737 (18.5)	204 (25.4)	
	≥60 to 100 (n/%)	15 (0.3)	2 (0.2)		10 (0.3)	4 (0.5)	
	Intoxication (n/%)	6 (0.1)	1 (0.1)		13 (0.3)	0 (0.0)	
**2012**							
Mean level (ng/mL)		23.4 ± 7.9	24.5 ± 7.9	<0.001	21.9 ± 8.9	23.2 ± 9.0	<0.001
	Deficient (n/%)	3,106 (33.8)	643 (29.5)		4,656 (85.6)	1,222 (81.7)	
	Sufficient (n/%)	6,082 (66.1)	1,534 (70.3)		765 (14.1)	269 (18.0)	
	≥60 to 100 (n/%)	10 (0.1)	4 (0.2)		14 (0.3)	3 (0.2)	
	Intoxication (n/%)	4 (0.0)	0 (0.0)		5 (0.1)	1 (0.1)	
**2013**							
Mean level (ng/mL)		24.1 ± 8.0	24.8 ± 7.6	<0.001	22.6 ± 8.8	24.1 ± 8.1	<0.001
	Deficient (n/%)	4,444 (30.4)	1,016 (25.8)		4,801 (83.6)	1,625 (78.8)	
	Sufficient (n/%)	10,151 (69.4)	2,917 (74.0)		921 (16.0)	431 (20.9)	
	≥60 to 100 (n/%)	22 (0.2)	10 (0.3)		13 (0.2)	6 (0.3)	
	Intoxication (n/%)	8 (0.1)	0 (0.0)		7 (0.1)	1 (0.0)	
**2014**							
Mean level (ng/mL)		28.4 ± 9.2	29.4 ± 8.7	<0.001	28.1 ± 9.6	29.0 ± 9.8	<0.001
	Deficient (n/%)	2,424 (15.2)	600 (10.9)		3,184 (62.6)	1,224 (58.4)	
	Sufficient (n/%)	13,432 (84.1)	4,877 (88.5)		1,859 (36.6)	853 (40.7)	
	≥60 to 100 (n/%)	107 (0.7)	32 (0.6)		35 (0.7)	17 (0.8)	
	Intoxication (n/%)	13 (0.1)	4 (0.1)		6 (0.1)	3 (0.1)	
**2015**							
Mean level (ng/mL)		28.6 ± 9.2	30.1 ± 9.3	<0.001	28.1 ± 9.8	29.9 ± 10.4	<0.001
	Deficient (n/%)	2,452 (14.6)	607 (9.4)		2,929 (61.3)	1,260 (55.4)	
	Sufficient (n/%)	14,277 (84.7)	5,809 (89.8)		1,812 (37.9)	988 (43.4)	
	≥60 to 100 (n/%)	100 (0.6)	45 (0.7)		28 (0.6)	25 (1.1)	
	Intoxication (n/%)	18 (0.1)	11 (0.2)		8 (0.2)	3 (0.1)	
**2016**							
Mean level (ng/mL)		28.6 ± 9.7	30.1 ± 10.1	<0.001	28.1 ± 10.0	30.1 ± 10.1	<0.001
	Deficient (n/%)	2,241 (15.0)	686 (11.2)		2,664 (63.4)	1,143 (55.8)	
	Sufficient (n/%)	12,605 (84.2)	5,382 (87.7)		1,499 (35.7)	882 (43.0)	
	≥60 to 100 (n/%)	103 (0.7)	60 (1.0)		36 (0.9)	23 (1.1)	
	Intoxication (n/%)	21 (0.1)	12 (0.2)		2 (0.0)	2 (0.1)	
**2017**							
Mean level (ng/mL)		29.0 ± 10.4	31.0 ± 8.3	<0.001	29.0 ± 9.7	30.6 ± 8.8	<0.001
	Deficient (n/%)	1,306 (9.6)	334 (5.6)		2,115 (55.7)	806 (44.2)	
	Sufficient (n/%)	12,180 (89.9)	5,617 (93.7)		1,665 (43.8)	1,006 (55.2)	
	≥60 to 100 (n/%)	47 (0.3)	32 (0.5)		15 (0.4)	10 (0.5)	
	Intoxication (n/%)	16 (0.1)	9 (0.2)		5 (0.1)	2 (0.1)	
**2018**							
Mean level (ng/mL)		27.9 ± 11.1	30.2 ± 11.0	<0.001	30.1 ± 72.2	29.5 ± 8.9	<0.001
	Deficient (n/%)	1,615 (13.6)	430 (7.8)		1,962 (61.0)	991 (56.4)	
	Sufficient (n/%)	10,245 (86.0)	5,044 (91.4)		1,217 (37.8)	758 (43.1)	
	≥60 to 100 (n/%)	40 (0.3)	40 (0.7)		30 (0.9)	8 (0.5)	
	Intoxication (n/%)	14 (0.1)	5 (0.1)		8 (0.2)	1 (0.1)	
**Total**							
Mean level (ng/mL)		27.2 ± 9.7	29.2 ± 9.6	<0.001	25.9 ± 23.2	28.0 ± 9.9	<0.001
	Deficient (n/%)	19,851 (19.1)	4,632 (12.5)		27,112 (70.9)	9,109 (62.0)	
	Sufficient (n/%)	83,401 (80.3)	32,074 (86.7)		10,837 (28.4)	5,471 (37.2)	
	≥60 to 100 (n/%)	472 (0.5)	229 (0.6)		214 (0.6)	101 (0.7)	
	Intoxication (n/%)	103 (0.1)	43 (0.1)		62 (0.2)	14 (0.1)	

M Mann-Whitney test
